# Oxidative Stress, Folate Receptor Autoimmunity, and CSF Findings in Severe Infantile Autism

**DOI:** 10.1155/2020/9095284

**Published:** 2020-11-18

**Authors:** Vincent T. Ramaekers, Jeffrey M. Sequeira, Beat Thöny, Edward V. Quadros

**Affiliations:** ^1^Center of Autism, University Hospital Liège (CHU), Liège, Belgium; ^2^Department of Medicine, SUNY-Downstate Medical Center, Brooklyn, New York, NY, USA; ^3^Division of Metabolism, University Children's Hospital, Zürich, Switzerland

## Abstract

**Background:**

Biomarkers such as oxidative stress, folate receptor alpha (FR*α*) autoimmunity, and abnormal brain serotonin turnover are common in autism.

**Methods:**

Oxidative stress biomarkers with pro- and antioxidants were measured in the severe form of infantile autism (*n* = 38) and controls (*n* = 24). Children and parents had repeated testing for serum FR autoantibodies, spinal fluid dopamine and serotonin metabolites, pterins, and N^5^-methyltetrahydrofolate (MTHF). Statistical analysis assessed correlations between variables. Genetic analysis included the SLC6A4 and SLC29A4 genes encoding synaptic serotonin reuptake proteins.

**Results:**

Compared to controls, the autism group showed a significant increase in oxidative DNA damage in lymphocytes, plasma ceruloplasmin and copper levels with a high copper/zinc ratio, thiol proteins, and superoxide dismutase (SOD) activity. Vitamin C levels were significantly diminished. In most autistic patients, the vitamin A (64%) and D (70%) levels were low. Serum FR autoantibodies fluctuating over 5–7 week periods presented in 68% of all autistic children, 41% of parents vs. 3.3% of control children and their parents. CSF showed lowered serotonin 5-hydroxyindole acetic acid (5HIAA) metabolites in 13 (34%), a low 5HIAA to HVA (dopamine metabolite) ratio in 5 (13%), low 5HIAA and MTHF in 2 (5%), and low MTHF in 8 patients (21%). A known SLC6A4 mutation was identified only in 1 autistic child with low CSF 5HIAA and a novel SLC29A4 mutation was identified in identical twins. Low CSF MTHF levels among only 26% of subjects can be explained by the fluctuating FR antibody titers. Two or more aberrant pro-oxidant and/or antioxidant factors predisposed to low CSF serotonin metabolites. Three autistic children having low CSF 5HIAA and elevated oxidative stress received antioxidative supplements followed by CSF 5HIAA normalisation.

**Conclusion:**

In autism, we found diverse combinations for FR autoimmunity and/or oxidative stress, both amenable to treatment. Parental and postnatal FR autoantibodies tend to block folate passage to the brain affecting folate-dependent pathways restored by folinic acid treatment, while an abnormal redox status tends to induce reduced serotonin turnover, corrected by antioxidant therapy. *Trial Registration*. The case-controlled study was approved in 2008 by the IRB at Liège University (Belgian Number: B70720083916). *Lay Summary*. Children with severe infantile autism frequently have serum folate receptor autoantibodies that block the transport of the essential vitamin folate across the blood-brain barrier to the brain. Parents are often asymptomatic carriers of these serum folate receptor autoantibodies, which in mothers can block folate passage across the placenta to their unborn child. This folate deficiency during the child's intrauterine development may predispose to neural tube defects and autism. Oxidative stress represents a condition with the presence of elevated toxic oxygen derivatives attributed to an imbalance between the formation and protection against these toxic reactive oxygen derivatives. Oxidative stress was found to be present in autistic children where these reactive oxygen derivatives can cause damage to DNA, which changes DNA function and regulation of gene expression. In addition, excessive amounts of these toxic oxygen derivatives are likely to damage the enzyme producing the neuromessenger serotonin in the brain, diminished in about 1/3 of the autistic children. Testing children with autism for oxidative stress and its origin, as well as testing for serum folate receptor autoantibodies, could open new approaches towards more effective treatments.

## 1. Introduction

Studies in autistic twins indicate involvement of both genetic and environmental factors. However, no common final pathway leading to autism spectrum disorders has been identified, apart from rare monogenetic, infectious, toxic, or environmental causes [1, 2]. The possible attribution by epigenetic factors came from postmortem brain tissue findings supporting a role of microRNA dysregulation in the pathophysiology of autism [3]. Recent studies have identified altered levels of salivary microRNA profiles in autism, differentiating autism spectrum disorders from peers with typical development and nonautistic developmental delay [4].

During the prenatal period, sufficient folate delivery to the developing embryo is necessary to prevent neural tube defects (NTDs) and autism spectrum disorders (ASDs) [5–7]. Despite normal maternal folate status during pregnancy, maternal serum FR*α* autoantibodies can impair folate passage across the placenta and predispose the fetus to NTD or autism [8, 9]. The prevalence of maternal FR*α* autoantibodies in families with autism varied between 26–59% while the prevalence of paternal FR*α* antibodies varied between 18–69% [9, 10]. Postnatal acquisition of FR*α* autoantibodies can cause infantile-onset cerebral folate deficiency (CFD) syndrome and autism spectrum disorders [11, 12]. The prevalence of FR*α* autoantibodies for autistic children varied between 47–76% [9, 10]. The prevalence of FR*α* antibodies in healthy adult women was estimated at 4–7% in Spain, 9–13% in Ireland, and 10–15% in the US population. A low titer of this antibody in a fraction of the adult population appears to be nonpathologic [9].

The cerebral folate deficiency (CFD) syndrome is defined as any neuropsychiatric condition with low CSF N^5^-methyltetrahydrofolate (MTHF), where FR*α* antibodies were identified in the majority of cases, while FOLR1 gene mutations or mitochondrial disorders remain rare alternative causes [12–15].

**Figure 1:**
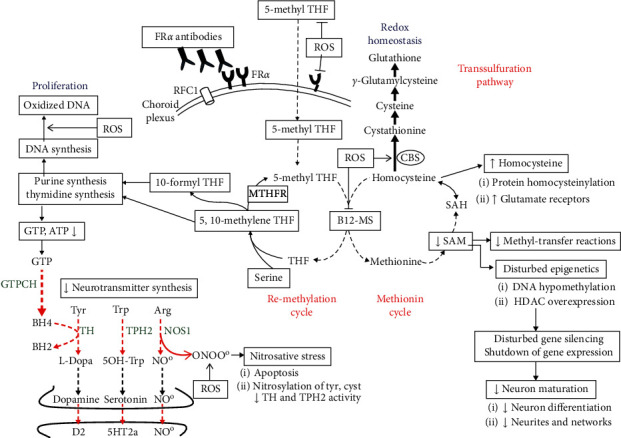
Pathophysiology of autism based on our findings showing the impact of reactive oxygen species (ROS) at different levels of intermediary metabolism and the consequences of brain 5-methyltetrahydrofolate (5-methyl THF) deficiency due to FR*α* autoimmunity. ROS inhibits B12-methionine synthase (B12-MS) activity and stimulates cystathionine beta synthase (CBS) activity, shifting the homocysteine accumulation from the methionine cycle into the transsulfuration pathway with increased production of the natural antioxidant glutathione. Superoxide anions also react with NO at the level of NO-synthase (NOS1) to form peroxynitrite instead of NO, which predisposes to apoptosis and nitrosylation of tyrosine and cysteine. Nitrosative stress affects activity of tryptophan (TPH2) and tyrosine hydroxylases (TH), the rate-limiting enzymes for serotonin, and dopamine synthesis. In addition, ROS catabolize 5-methyl-THF and impair folate uptake and transcellular transport across the choroid plexus and placental barriers due to interaction with FR*α* and RFC1 folate transporters. FR*α* autoantibodies also impair folate transport to the fetus and brain. The resulting brain folate deficiency predisposes to reduce SAM production and SAM-dependent methyl-transfer reactions and reduces purine and thymidine synthesis with diminished GTP and BH4 production. The diminished BH4 availability as the shared cofactor of the enzymes TH, TPH2, and NOS1 will therefore reduce their enzyme activity. Reduction of the activated methyl group donor SAM downregulates DNA methylation and affects posttranslational modifications of histones (methylation and trimethylation of histones), thereby impeding the homeostatic balance between gene transcription and silencing. In addition, folate deficiency is accompanied by overexpression of histone deacetylases, which further leads to abnormal gene silencing. The shutdown in expression of specific sets of genes will affect neuronal growth, pruning, and differentiation. Abbreviations: GTPCH: GTP-cyclohydrolase I; Arg: arginine; Cyst: cysteine; Tyr: tyrosine; Trp: tryptophan; MTHFR: methylenetetrahydrofolate reductase; RFC1: reduced folate carrier-1 (reproduced with permission from [20]).

Based upon findings in mitochondrial encephalopathies, Kearns–Sayre syndrome, and Alper's disease, reactive oxygen species (ROS) and low ATP production were suspected to reduce MTHF stability and impair folate transport to the brain. In vitro studies showed that in KB cells (ubiquitous keratin-forming tumor cell line HeLa), expressing the FR*α* and the reduced folate carrier (RFC1), MTHF uptake was impaired following exposure to reactive oxygen species (ROS). This could be prevented by the radical scavenger ascorbic acid as an antioxidant [16]. In patients with mitochondrial encephalopathies, the imbalance between ROS formation and antioxidant defenses causes oxidative stress and damage to reduced folates and to folate transporter proteins [16], explaining the instability of reduced folates and disruption of folate transfer across the blood-CSF barrier. Although evidence is limited in autism, prior studies have suggested increased vulnerability to oxidative stress and decreased methylation capacity in its pathogenesis [17–19]. The different effects exerted by ROS and the nitric oxide (NO) derived peroxynitrite radical (nitrosative stress) upon one-carbon metabolism and passage of MTHF across placenta and blood-brain barriers as well on DNA damage are explained by Figure 1. ROS inhibit the methionine synthase activity and remethylation cycles. Simultaneously, ROS stimulate cystathionine beta synthase activity shifting homocysteine from the methionine cycle into the transsulfuration pathway, thereby increasing glutathione production. Increased ROS favour DNA oxidation, while superoxide radicals promote peroxynitrite formation with consequent nitrosative stress. The consequences of blocked folate passage to the CNS, the impact by ROS and nitrosative stress upon one-carbon metabolism, DNA stability, neurotransmitter synthesis, and NO production are depicted in Figure 1.

**Figure 2:**
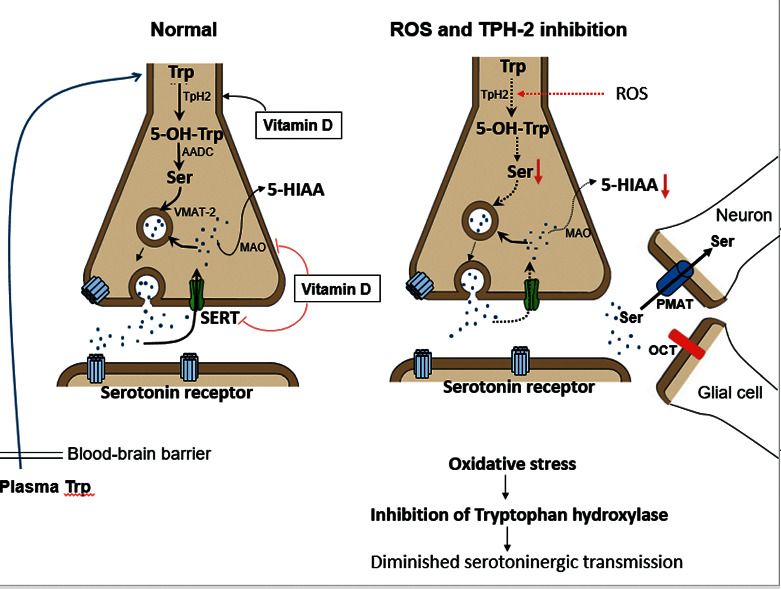
On the left side, the normal intestinal absorption, passage across the blood-brain barrier, and neuronal Trp uptake are shown. Neuronal Trp will be hydroxylated by tryptophan hydroxylase to 5-hydroxytryptophan (5-OH-Trp) and decarboxylated by aromatic amino acid decarboxylase to serotonin, which is then transported by the vesicular monamine transporter (VMAT-2) into presynaptic vesicles. After serotonin releases into the synaptic cleft, part of the serotonin pool will be cleared from the synaptic space by SERT into the presynaptic compartment where it will be partly recycled into vesicles or catabolized by MAO to 5HIAA. Another part of the synaptic serotonin pool will be taken up by PMAT-expressing neurons or OCT-expressing glial cells. On the right, the findings of this study suggest diverse causes contributing to lowered serotonin turnover. Abundant formation of ROS or peroxynitrite probably represents a major factor involving TPH2 enzyme dysfunction through oxidation of cysteine rich sites at the Trp binding site. In a minority of cases, a low plasma Trp as substrate for serotonin (5HT) production may be due to low dietary Trp intake or malabsorption. Rare genetic mutations of the gene encoding the serotonin reuptake transporter (SERT) lead to a gain of function of serotonin reuptake leading to diminished serotonin availability at postsynaptic receptors. Other rare mutations affect the gene encoding PMAT involving reduced clearance of serotonin from synaptic spaces, thereby disturbing serotonin turnover during early fetal brain development.

Brain PET scan studies using the radioactive serotonin precursor *α*[11C]-methyl-L-tryptophan indicated diminished whole brain or regional serotonin levels in autistic children compared to controls [21]. However, the compromised brain serotonin status remains an enigma. After transport of the amino acid precursor L-tryptophan (Trp) across the blood-brain barrier and its uptake by specific serotonin producing raphe neurons, the first rate-limiting step of serotonin synthesis is mediated by the neuronal enzyme tryptophan hydroxylase 2 (TPH2). 5-Hydroxytryptophan (5OH-Trp) will be decarboxylated by the vitamin B6-dependent enzyme aromatic amino acid decarboxylase to serotonin (5HT; 5-hydroxytryptamin). Serotonin is then packaged by the action of vesicular monoamine transporter isoform 2 (VMAT-2). Vesicular serotonin is released at axonal terminals into the synapse for subsequent action at postsynaptic serotonin receptors. Serotonin is cleared from the synaptic cleft through action of the serotonin reuptake transporter (SERT) into the presynaptic space and partly through two other serotonin reuptake transporters located in neurons and glial cells surrounding the synapse, being the plasma monoamine transporter (PMAT) and the organic cation transporter (OCT). After re-entry into the presynaptic space, part of the serotonin pool will be recycled into vesicles and part will be catalyzed by monoamine oxidase-A (MAO-A) and aldehyde dehydrogenase to the serotonin end-metabolite 5-hydroxyindoleacetic acid (5HIAA). The three most important enzymes and transport proteins that determine serotonin homeostasis and turnover in the CNS are TPH2, SERT, and MAO-A (Figure 2).

Clinical anecdotal reports and animal and genetic studies in ASD have addressed the serotonin enigma. In comparison with healthy age-matched controls, a previous spinal fluid study among 97 ASD patients confirmed low CSF levels for the serotonin end-metabolite 5-hydroxyindole acetic acid (5HIAA) levels among 31% of the patients and low MTHF levels in 21% of patients, while 48% of all patients had normal CSF findings [22]. A genetic study on 248 ASD patients including the 97 patients having the CSF analysis found a p.Gly56Ala mutation in the SLC6A4 gene with a gain of function of the SERT protein at an equal frequency in the ASD and control populations. In contrast, among the 248 ASD patients, a prevalence (3.2%) was detected for three novel heterozygous nonsynonymous mutations within the SLC29A4 plasma monoamine transporter gene (PMAT), revealing significantly reduced reuptake activity towards a variety of substrates including serotonin, dopamine, and 1-methyl-4-phenylpyridinium (MPP^+^). However, these genetic studies linking SLC29A4 gene mutations encoding PMAT to the ASD population did not always coincide with low CSF 5HIAA metabolites. PMAT dysfunction in ASD subjects was speculated to raise serotonin prenatally, exerting a negative feedback inhibition through serotonin receptors on development of serotonin networks and local serotonin synthesis.

Despite the known heterozygous p.Gly56Ala mutation in the SLC6A4 gene, found in the ASD and control populations, a recent study in a mouse model showed a substantial impact of the maternal SLC6A4 (SERT) homozygous p.Gly56Ala genotype on early placental serotonin levels, forebrain serotonin levels, and neurodevelopment [23].

The link between brain hyposerotonin and autism was also supported by the neuronal TPH2 gene knockout mouse model predisposing to lowered brain serotonin production [24]. However, the contribution of genetic factors affecting serotonin in ASD remains weak. Other nutritional factors like vitamin D status have been shown to play a role in causing brain hyposerotonin because vitamin D is involved in transcriptional regulation of neuronal TPH2, SERT, and monoamine oxidase-A (MAO-A) governing serotonin homeostasis [25, 26]. Vitamin D acts through vitamin D response elements (VDREs) at these genes through activation of TPH2 gene transcription and simultaneous inhibition of SERT and MAO-A gene expression. Therefore, vitamin D deficiency predisposes to low serotonin production and increased reuptake and catabolism of serotonin in the brain. Data supporting an increased risk for autism in the offspring due to maternal intake of selective serotonin reuptake inhibitors have remained inconclusive [27].

To address these unresolved issues of serotonin metabolism, the objective of this case-controlled study in infantile autism was to analyze simultaneously CSF neurotransmitter and folate metabolites and serum FR*α* antibodies in children and their parents and to identify the role of oxidative stress with elevated pro-oxidants or failing antioxidant defenses. In addition, we analyzed vitamin A and D levels because these vitamins are putative antioxidants and vitamin D plays a role in maintaining serotonin homeostasis and folate transport across the blood-brain barrier [28]. The analysis of our data of this study examined a postulated link between FR*α* autoantibodies and oxidative stress on one hand and on the other hand deranged folate transfer to the brain, as well as deranged brain neurotransmitter metabolism, in particular serotonin turnover (see Figure 2).

## 2. Patients and Methods

### 2.1. Participants

The participants recruited for this study were children referred to our center of autism for diagnosis and treatment. Investigations included the ADI-R and ADOS tests, Childhood Autism Rating Scale (CARS), development and speech assessment, psychological and psychiatric assessment, and observational evaluation at school or in the domestic situation. The CARS assessment at baseline performed in 38 autistic patients showed a severe form of infantile autism in the majority of 34 patients (CARS between 37 and 60; mean ± SD: 43.76 ± 7.11) and mild to moderate autism in 4 patients (CARS between 30 and 36.5).

Each patient had a complete history, physical and neurologic examination, brain MRI, and prolonged EEG recording. Other investigations included metabolic screening and measuring urinary amino acids and organic acids, creatine, and guanidinoacetate excretion. Genetic testing included Angelman syndrome, fragile X-syndrome, the MECP2 gene mutations, chromosome analysis, and array CGH to detect microdeletions or microduplications. In total, we only recruited 38 patients older than 3½ years, diagnosed with nonsyndromic infantile autism after exclusion of brain abnormalities, intractable epilepsy, and metabolic and recognizable genetic abnormalities or syndromes. In the autism group, all parents were healthy without a neurologic or psychiatric history. Other inclusion criteria for the autistic children comprised the absence of a special diet, any medication or supplements, and finally no specific behavioral intervention at the beginning of this study. The control group consisted of 24 age-matched healthy children and parents.

### 2.2. Design of Case-Control Study on Oxidative Stress, FR Autoimmunity, and CSF Metabolites

The study was approved by the University Hospital Liège Ethics Committee and the IRB at SUNY Downstate Medical Center, New York. Children with infantile autism (*n* = 38) and healthy controls (*n* = 24) were at least 3½ years old. After parental informed consent and overnight fasting, each autistic child or healthy control had blood samples taken for oxidative stress parameters and serum FR*α*-autoantibodies while simultaneously a spinal tap was performed for the autistic children. For both groups, blood was drawn for measurement of oxidative stress parameters as well as pro-oxidant and antioxidant factors (see below). Results obtained in the autism group were compared with the healthy age-matched control group by statistical analysis (see below). Serum from patients was analyzed for FR*α*-autoantibodies of the blocking and binding type and compared to healthy controls. Parents of both groups were asked to participate in the serum FR*α* antibody testing. On follow-up, serial samples for detection of FR*α* antibodies were drawn at one-week intervals in 6 children with autism, including one monozygotic twin pair. Following the spinal tap, we analyzed cerebrospinal fluid (CSF) biogenic monoamine metabolites, pterins, and MTHF. For each autistic child, we also determined fasting plasma amino acids.

### 2.3. Oxidative Stress and Pro-Oxidant and Antioxidant Biomarkers

In the present case-control study, oxidative stress status was investigated by measuring oxidative damage to lipids and DNA and levels of natural pro-oxidants, antioxidants, and free radical scavengers (supplement 1). Biomarkers for oxidative stress were lipid peroxides, oxidized LDL, and antibodies against oxidized LDL proteins, while the comet assay measured the percentage of DNA damage by detecting single/double-strand DNA breaks, alkali labile sites (apurinic/apyrimidinic sites), DNA cross-links, base/base-pair damages, and apoptotic nuclei in lymphocytes [29]. For the antioxidant defenses, we measured paroxonase, total and oxidized glutathione, thiol proteins, ceruloplasmin, apolipoprotein-B, vitamins C and E, ubichinon-Q10, gamma-tocopherol, beta-carotene, the antioxidant enzyme glutathione peroxidase and its co-factor selenium, and superoxide dismutase with its different enzyme co-factors copper, zinc, and manganese [30–35]. We also determined serum iron and ferritin as co-factors of the antioxidant enzyme catalase. As putative antioxidants, we also included measurement of vitamin A and D levels.

For the pro-oxidant factors, we measured beta-carotene, iron, copper, zinc, and the copper-zinc ratio as well as manganese because elevated values of these metals and trace elements or an increased copper/zinc ratio act to promote oxidative stress and the Fenton reaction to generate hydroxyl radicals [36]. In this context, it should be stressed that some factors like beta-carotene possess a dual function acting as antioxidant at physiological concentrations but turning into a pro-oxidant at elevated concentrations [37].

For each child, the following routine measurements have also been included: complete blood count (CBC), serum and RBC folate, vitamin B_12_, renal and liver function, thyroid function (TSH, T3, and T4), lactate, CPK, alkaline phosphatase, calcium, magnesium, and cholesterol. Serum samples were used to measure anti-gliadin antibodies.

### 2.4. Serum FR*α* Autoantibodies

The assays for both the blocking and binding FR*α* autoantibodies have been described previously. Blocking FR*α* autoantibodies in serum were expressed as pmoles of radioactive folic acid blocked from binding to FR*α*-antigen per ml of serum and binding FR*α* autoantibodies were expressed as pmoles of IgG antibody per ml of serum [38].

### 2.5. CSF Analysis

After overnight fasting, CSF samples were collected by lumbar puncture, and measurements included cells, protein and glucose, the intermediary metabolites (L-dopa, 3-O-methyl-dopa, and 5OH-Trp) and end-metabolites of dopamine and serotonin, i.e., homovanillic acid (HVA) and 5-hydroxindole- acetic acid (5HIAA), the pterin metabolites neopterin and biopterin, and N^5^-methyltetrahydrofolate (MTHF). The HVA/5HIAA ratio was calculated. Biogenic monoamine metabolites have been measured by a HPLC method. Total neopterin and biopterin (representing the sum of tetra- and dihydro-biopterin and biopterin) were measured by HPLC after oxidation with manganese dioxide. MTHF was measured with HPLC using electrochemical detection. Separation was achieved on a 5*μ* Sperisorb ODS-1 (250 × 4.6 mm) analytical column (Stagroma AG, Switzerland), using a 50 mM sodium acetate in 22% (v/v) methanol, pH 4.5 as a mobile phase. The flow rate was 1 ml/min, and the analytical cell (Model 5011, ESA, Bedford, MA, USA) was adjusted to +0.20 V (ESA Coulochem Model 5100A, ESA) with a response time of 2 s. [39–41].

CSF data were compared to previously collected samples from healthy children classified according to different age groups at the University Children's Hospital, Zürich [39–41]. Because the focus in autism is on reduced serotonin turnover, we performed appropriate testing of SLC6A4 and SLC29A4 genes encoding the SERT and PMAT proteins, as described previously [22].

For a selected group of children with oxidative stress and low serotonin metabolites in CSF, the biochemical and clinical evolution was followed after antioxidant treatment as outlined in a previous study and by supplement 2 and after approval by the ethics committee and informed consent [20].

### 2.6. Statistical Analysis

The measured oxidative stress biomarkers and pro- and antioxidants were compared between the group of autism and healthy age-matched controls using the unpaired *t*-test.

Within the autism group, statistical analysis evaluated correlation coefficients between on one hand the CSF findings and on the other hand the biomarkers for oxidative stress, each of the pro-and antioxidant parameters, FR autoantibody titers, and the routine parameters iron, ferritin, and vitamins A and D.

In addition, statistical analysis evaluated the cumulative number of aberrant pro- and antioxidant parameters present for each autistic individual compared to each of the oxidative stress biomarkers and also to the distribution of normal and abnormal CSF findings for MTHF and biogenic monoamine metabolites HVA and 5HIAA.

Further statistical analysis used the chi-square test to validate the different hypotheses evoked by our findings.

## 3. Results

### 3.1. Oxidative Stress Biomarkers and Pro- and Antioxidant Factors

Thirty-eight patients with infantile autism participated in this study. Their age was 7.25 ± 3.9 years (mean ± SD) with an age range between 3.6–18 years. The male/female sex ratio was 4.4 : 1. For the 24 age-matched controls, there was an equal gender distribution and compared to the 38 autistic patients, there was no significant difference with respect to their age of 8.7 ± 3.2 years that ranged from 4.4–16 years.

Table 1 shows the results of oxidative stress parameters between the infantile autism group (*n* = 38) compared to the healthy control group (*n* = 24). The statistical test was the unpaired *t*-test. Compared to controls, the comet assay in lymphocytes was the only biomarker for oxidative stress to show significantly increased values for oxidized DNA, whereas the other oxidative stress parameters, i.e., plasma lipid peroxides, oxidized low-density lipoprotein (LDLox), and antibodies against oxidized LDL, did not differ between the autistic and healthy control groups. Although the zinc values in the autistic group remained within the normal range, the copper and ceruloplasmin values for the autistic group were significantly higher compared to the control group. As a consequence, the copper/zinc ratio became significantly elevated for the autistic group. Among all measured antioxidant parameters, the vitamin C values were significantly lowered for the group with autism vs. controls. Although total glutathione and oxidized glutathione levels were not different, total thiol-protein levels were significantly higher for the autism group. In addition, the values for superoxide dismutase activity in autism were significantly raised. Although the putative antioxidants vitamins A and D were not considered among the antioxidants in this study, the autistic group had a high prevalence of lowered values for the putative antioxidants vitamin A (64%) and vitamin D (70%). Other parameters showed no statistical difference between autism and controls.

Although there was no consistent statistical difference for each of all the measured parameters (oxidative stress biomarkers and antioxidant and pro-oxidant factors) between the whole group of autistic patients vs. healthy controls, 25 of the 38 autistic children (66%) had one or a combination of two elevated oxidative stress biomarkers (oxidized DNA in lymphocytes above the upper boundary of the reference range for healthy controls among 12 autistic patients, elevated lipid peroxides in 2 patients, high oxidized LDL in 7 patients, and high antibodies against oxidized LDL in 11 patients). In 20 of these 25 autistic patients with one or two elevated oxidative stress biomarkers, we found the presence of one to 4 pro- or antioxidant parameters outside the normal reference range, defined as values above the upper boundary or below the lower boundary of the range in healthy controls (*n* = 24). However, among 5 out of 25 autistic children with elevated oxidative stress biomarkers (oxidized DNA damage in 2 patients, combined oxidized DNA and oxidized LDL in 1 patient, combined oxidized DNA and antibodies against oxidized LDL in 1 patient, and combined elevated values for oxidized LDL and antibodies against LDL in 1 patient), all measured pro- and antioxidant factors were normal (data not shown). In these latter 5 patients, other pro-oxidant factors predisposing to oxidative stress, like heavy metal poisoning (aluminium, lead, and mercury), increased manganese, copper/zinc ratios, or beta-carotene, were excluded.

However, among the 38 autistic patients, there was a random distribution of abnormal pro-oxidant and/or antioxidant factors without a clear association between any specific factor and the different oxidative stress biomarkers. Eight of the 38 autistic patients had normal pro- and/or antioxidant parameters, whereas there were 9 patients having only one abnormal pro-oxidant or antioxidant parameter, 9 patients with a combination of two, 7 patients with three, and finally 5 patients with four aberrant pro- and/or antioxidants. Among all 30 autistic patients with abnormal pro- and/or antioxidants, the pro-oxidant factors included elevated selenium (6 patients) and zinc (5 patients), an increased copper/zinc ratio (4 patients), and elevated beta-carotene (1 patient). The antioxidant factors in decreasing frequency were low iron (9 patients), diminished apolipoprotein-B (7 patients), low total glutathione (6 patients), low gamma-tocopherol (5 patients), low manganese (5 patients), lowered enzyme activity for plasma and red blood cell glutathione peroxidase (5 patients), low selenium (4 patients), low vitamin C levels (4 patients), lowered ferritin levels (3 patients), low Q10 (2 patients), low beta-carotene (2 patients), and lowered vitamin E levels (1 patient).

We compared each oxidative stress biomarker from the autism group as a function of the total number of abnormal pro- and/or antioxidants with the oxidative stress biomarker values obtained for the healthy control group. Compared to the control group, the comet assay (oxidized DNA) for autistic children was significantly increased not only for the subgroup having normal pro- and antioxidants but also for each of the subgroups' autistic children having only 1 abnormal pro- or antioxidant parameter or a combination of 2, 3, or 4 simultaneously measured abnormal pro- and/or antioxidant parameters. Although numbers are too small to reach statistical significance, comparison between the subgroup autistic patients with oxidized DNA within the normal range and the subgroup with elevated oxidized DNA above the upper boundary of the healthy control range demonstrated that the subgroup with elevated oxidized DNA tended to have low values for the antioxidant enzyme co-factors manganese and selenium, total glutathione, gamma-tocopherol, and vitamin E.

The oxidative biomarker lipid peroxides did not show a significant difference between the whole autistic group vs. the healthy control group. In the autistic group, there were only two patients having an elevated lipid peroxide value above the upper boundary of the healthy control range. However, the lipid peroxide levels from the subgroup autistic patients having 2 or more abnormal pro- and/or antioxidant values (mean ± SD: 774 ± 349 *μ*M; *n* = 21) were significantly higher in comparison with the lipid peroxides for the subgroup of autistic patients with either normal or only one abnormal pro- or antioxidant parameter (mean ± SD: 549 ± 290 *μ*M; *n* = 17; *t* = 2.12; *p* = 0.04). Compared to controls, lipid peroxide levels for the autism subgroup having normal or only one abnormal value for pro- or antioxidant parameters did not show statistical difference. However, the lipid peroxide levels for the autistic subgroup (*n* = 12) having a cumulative sum of 3 or 4 abnormal pro- and antioxidants (mean ± SD: 888 ± 335 *μ*M) were significantly higher compared to healthy controls (*n* = 24; mean ± SD: 609 ± 219 *μ*M; *t* = 3; *p* = 0.004).

The other oxidative stress biomarkers oxidized LDL and antibodies against oxidized LDL did not show statistically significant differences between the autism and healthy control groups. Within the autism group, the latter oxidative stress biomarkers did not change significantly as a function of the cumulative number of abnormal pro- and antioxidant parameters.

### 3.2. FR*α* Autoantibodies

**Figure 3:**
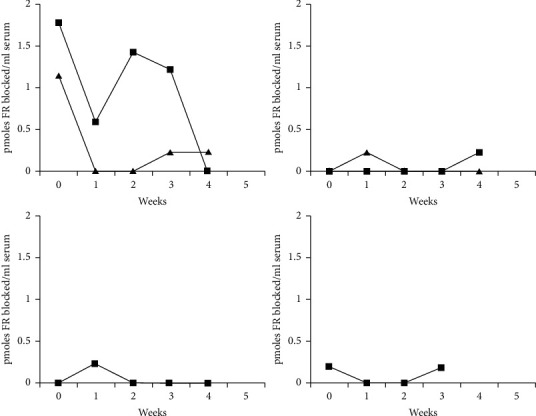
Fluctuations of titers for FR antibodies of the blocking type over 5 weeks in five patients with autism, with the upper left side showing two patients with high fluctuating FR antibodies of the blocking type and the upper right side showing lower FR antibody fluctuations in the identical twin pair. Both curves at the lower part represent two other patients having a minor FR antibody peak over a period of 4 to 5 weeks.

Compared to FR*α* antibodies of the blocking type in only 1/30 controls, these were present in 26/38 autistic patients (68%) (chi-square test at 30.12 with *p* < 0.001). Upon initial testing, two autistic patients tested negative but had positive FR*α* autoantibodies upon follow-up testing. Among 6 patients, whose serum samples were collected at 1-week intervals, fluctuating FR*α* antibody titers varying from nondetectable levels to high titers were found in two patients, whereas a minor peak of FR antibodies occurred once during five weeks in four patients, including one identical twin pair (Figure 3). Among 26 out of 38 autistic children, the detection of positive FR*α* autoantibodies of the blocking type (mean ± SD for all 38 patients: 0.59 ± 1.17 pmol FR*α* antigen blocked/ml serum) was accompanied by the simultaneous presence of FR*α* autoantibodies of the binding type among 6 out of 17 tested individuals (mean ± SD: 0.30 ± 0.51 pmol IgG/ml serum). In only 1 autistic child, FR*α* autoantibodies of the binding type could be detected in the absence of FR*α* antibodies of the blocking type. Among 29 out of 38 families, data were available for FR*α* antibodies in the child and both parents (Table 2). In this group of 29 families, FR*α* antibodies of the blocking type (mean ± SD: 0.62 ± 0.63 pmol FR*α* antigen blocked/ml serum) were positive in 20 children from 29 families (69%) with simultaneous presence of FR*α* antibodies of the binding type in 5 out of 15 tested individuals (mean ± SD: 0.73 ± 0.61 pmol IgG/ml serum), while only 1 child from these 29 families had FR*α* antibodies of the binding type, but no blocking FR*α* antibodies. Testing of parents of autistic children available from these 29 families showed that 12 parents out of 29 families (41%) tested positive, including either the mother (5/29), the father (3/29), or both parents (4/29). In contrast, among the control group of 30 healthy nonautistic children, we only detected FR antibodies in 1 child and his parents (3.3%). Statistical analysis indicated a high association of parental FR*α* antibodies in the autism group (chi-square test at 14.26 with *p* < 0.001).

### 3.3. Spinal Fluid Findings

Normal CSF results were found in 10/38 (27%) of the patients, and abnormal CSF results were found in the other 28 patients (73%), as shown in Table 3. In 13/38 patients (34%), 5HIAA levels in spinal fluid were lowered. Although 5 other patients (13%) had 5HIAA values remaining within the normal range, the levels remained relatively decreased compared to the HVA values as illustrated by higher HVA/5HIAA ratios above 3.5 (normal range of HVA/5HIAA ratio from 1.5–3.5). In 2 patients (5%), both 5HIAA and MTHF concentrations were lowered, while in eight patients (21%), only the CSF MTHF level was lowered.

Considering the presence or absence of oxidative stress and FR*α* autoantibodies, four different groups of patients could be distinguished. Eighteen patients had both elevated oxidative stress and FR autoantibodies, which resulted in low 5HIAA in 8 patients, low CSF MTHF in 3 patients, and normal results in 7 patients. In 7 other patients, there was only evidence for oxidative stress, which was accompanied by either low CSF MTHF (1 patient), combined low MTHF and 5HIAA (1 patient), and decreased or relatively low 5HIAA levels in 5 patients. FR*α* autoimmunity without oxidative stress in 6 patients was associated with either low CSF MTHF in 3 patients, low 5HIAA in 2 patients, or normal findings in 1 patient. In 7 patients with absent FR*α* autoantibodies and no elevated oxidative stress biomarkers, abnormalities of CSF metabolites were still encountered, except in 2 patients with normal CSF findings. Among several patients having follow-up for oxidative stress biomarkers, fluctuations of these biomarkers and the pro- and antioxidant parameters were noticed. Fasting plasma amino acids showed low Trp values in 2 patients with low CSF 5HIAA levels, while Trp values for the other patients remained within the normal range.

**Figure 4:**
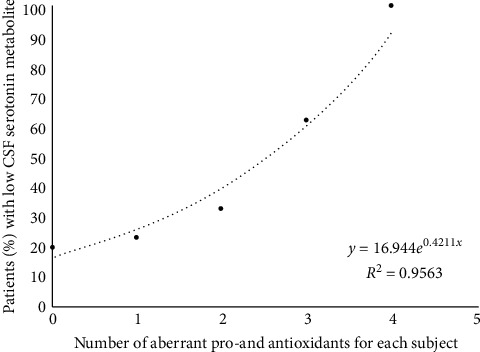
The relationship between the serotonin metabolite 5HIAA concentration and redox status. This plot shows the cumulative number of antioxidant deficiencies or increased pro-oxidants on the *X*-axis and the percentage of patients with low CSF 5HIAA metabolites on the *Y*-axis, representing the end metabolite of serotonin production.

The absence of an abnormal redox status or an average number of 1 aberrant value for pro- and/or antioxidants was associated with either normal CSF profiles (38–40%), low CSF MTHF (30%), low CSF 5HIAA (20–23%), or combined lowering of 5HIAA and MTHF (7–10%). An average number of two or more than two aberrant pro-oxidant and/or antioxidant parameters tended to be associated with a higher prevalence of children with low CSF 5HIAA values (Figure 4). Statistical analysis confirmed a significant higher proportion of low CSF 5HIAA levels among children with two or more than two aberrant pro- and/or antioxidant parameters compared to the group of children with a normal redox status or only 1 abnormal pro- and/or antioxidant parameter (chi-square test at 4.55 with *p* < 0.05).

Further statistical analysis did not find any other correlation between the values of measured CSF metabolites and oxidative stress biomarkers, pro- and antioxidant values, serum and RBC folate, iron and ferritin, vitamins A and D, and serum FR autoantibody titers. In addition, no correlation was found between the measured titers of FR*α* autoantibodies and oxidative stress biomarkers, pro- and antioxidant parameters, and vitamins A and D.

### 3.4. Genetic Findings

Genetic testing in identical twins with severe autism from consanguineous first-line cousins whose CSF findings were normal showed the presence of a heterozygous *SLC29A4* gene mutation (c.412G>A mutation resulting in a p.Ala138Thr change), encoding a functional loss for the plasma membrane monoamine transporter (PMAT). The association of PMAT mutations with autism was previously reported by our group [22]. In one patient with low CSF 5HIAA, a common heterozygous *SLC6A4* gene Gly56Ala mutation encoding the serotonin reuptake transporter protein (SERT) was detected. This led to a gain of function as reported previously in another patient [42]. The heterozygous SERT mutation is found at equal frequency in autism and healthy controls. A 2q22.2-q23.3 microduplication was found in another patient with low 5HIAA levels in CSF, and this microduplication has been described in the literature to be associated with developmental delay and autism [43]. An earlier described Xp22.31 microduplication was found in one patient with an increased HVA/5HIAA ratio and has been reported by others [44]. In one patient with normal CSF data, we found a recently reported 15q11.2 microdeletion [45].

### 3.5. Effect of Antioxidant Supplementation on CSF Abnormalities

After identification of low CSF 5HIAA and oxidative stress in 3 patients of whom 2 had FR*α* antibodies, supplements with antioxidants were administered during 8 (patients 1 and 2) or 24 months (patient 3), respectively. Follow-up spinal tap of these 3 patients showed normalisation of previously low 5HIAA values (Table 4). The youngest 3½-year-old autistic child was positive for serum FR*α* autoantibodies at low titers which were also present in the mother but absent in the father. Oxidative stress biomarkers showed increased lipid peroxides at 1283 *μ*M (control group mean ± SD: 609 ± 219; range: 183–1095 *μ*M) associated with low serum apolipoprotein-B at 0.38 g/L (control group range: 0.60–1.40), low vitamin A and vitamin E, and low glutathione, selenium, and zinc. CSF showed low 5HIAA at 104 nM and normal HVA values with an elevated HVA/5HIAA ratio of 5.4 (normal 1.5–3.5) and normal findings for MTHF and pterins. Daily supplements during 8 months of vitamin C, vitamin E, vitamin A, selenium, and zinc sulfate resulted in normal CSF findings 10 months later. The child showed improved nonverbal communicative skills and social interaction. His CARS score at baseline was compatible with severe autism (CARS 42), and following supplements during 8 months, his CARS decreased towards 34, reflecting moderate autism. The second child was 4½ years and had no serum FR*α* antibodies, whereas his mother tested positive for FR*α* antibodies. Oxidative stress included increased lipid peroxides at 1790 *μ*M (control group mean ± SD: 609 ± 219; range: 183–1095 *μ*M) attributed to low co-enzyme Q10, elevated copper and an increased copper/zinc ratio at 2.34 (normal range: 0.70–1.66), low vitamin A, and low normal selenium with decreased selenium-dependent glutathione peroxidase activity. CSF showed diminished 5HIAA at 81 nM (normal range: 105–299) and normal HVA with an increased HVA/5HIAA ratio at 4.2 (normal range: 1.5–3.5) in the presence of normal MTHF and pterins. Daily supplements of co-enzyme Q10, selenium, and zinc sulfate during 8 months resulted in normal lipid peroxides at 945 *μ*M and normal CSF 5HIAA levels. Clinical amelioration included improved verbal and nonverbal communication, first indications of social interaction, disappearance of agitation, and better concentration. However, his CARS score of 45.5 at baseline only diminished slightly towards 43.5. The third patient was 17 years old at the time of investigation and tested positive for FR*α* antibodies, while both parents tested negative. Oxidative stress biomarkers showed increased oxidized DNA at 29% (control group mean ± SD: 14.9 ± 3.9; range: 10.2–21.5) attributed to low serum manganese, co-enzyme Q10, and ferritin values. Plasma L-Trp was low at 15 *μ*M (normal range: 27–75). CSF analysis showed lowered 5HIAA and HVA values with normal MTHF. After he received supplements with iron, vitamin C, vitamin E, and Q10 during 2 years, the result of repeated CSF analysis 2½ years later showed an increase and normalisation of CSF 5HIAA and HVA levels. However, there was minimal clinical amelioration as was reflected by no significant change of the severity of autism. The patient's older age of 17 years represents a possible negative factor explaining the poor clinical response despite CSF normalisation.

## 4. Discussion

This study suggests that oxidative stress and FR*α* autoimmunity represent two independent parameters capable of affecting DNA integrity, brain neurotransmitters, and folate-dependent one-carbon metabolism, disrupting epigenetic programming. Thus, not only treatment for FR*α* autoimmunity has to be taken into account [13], but also specific interventions to neutralize abundant reactive oxygen species (ROS) which can derange folate homeostasis, serotonin turnover, intermediary brain metabolism, DNA integrity, and epigenetics at different levels should be considered. The multiple points where oxidative or nitrosative stress perturb intermediary metabolism, folate transport, and genetic integrity have been summarised by Figure 1. A recent treatment trial for autism confirmed a positive effect on outcome by a combined therapeutic approach that aimed to neutralize oxidative stress and alleviate FR*α* autoimmunity [45].

In previous *in vitro* studies, we found that the generation of superoxide anions *in vitro* catabolizes MTHF by 75% within one hour, which can be prevented by the radical scavenger ascorbic acid [16]. After exposure to superoxide anions in culture, cellular folate incorporation was reduced in KB cells expressing FR*α* and RFC1 proteins. Thus, transmembrane MTHF passage at the placenta and choroid plexus is likely to be impaired by ROS, which leads to fetal and cerebral folate deficiency. ROS will react with NO-producing neurons and lead to peroxynitrite formation within these neurons, which can lead to neuronal dysfunction and eventually apoptosis [46, 47].

In addition to *in vivo* superoxide anion generation promoting peroxynitrite formation (Figure 1), additional neuronal folate depletion lowers purine synthesis and thus the pool of guanosine triphosphate as substrate for GTP-cyclohydrolase I, the rate-limiting enzyme for tetrahydrobiopterin (BH4) production [48]. Since BH4 acts as common co-factor for tryptophan- and tyrosine-hydroxylase and NO-synthase, folate depletion will limit serotonin, dopamine, and NO production. Moreover, in the presence of low BH4, NO-synthase shifts its enzymatic activity and, instead of NO formation, starts to produce the nitrosyl radical peroxynitrite [49]. Moreover, peroxynitrite inactivates tryptophan- and tyrosine-hydroxylases via sulfhydryl oxidation at their enzyme substrate-binding site, containing cysteine residues [50, 51].

Peroxynitrite also nitrosylates enzymatic protein tyrosine residues, but this exerts a minimal effect [49]. In addition, oxidation induces neuronal TPH2 aggregates through disulfide cross-linking [50]. Thus, generation of ROS and peroxynitrite, enhanced by folate depletion, reduces production of dopamine, NO, and in particular serotonin, as confirmed by previous findings of low serotonin production among 1/3 of autistic patients [21, 22]. The findings of the present study confirm that the cumulative presence of multiple pro-oxidant factors and/or antioxidant deficiencies present in each individual increases the percentage of patients with low CSF serotonin metabolite 5HIAA (Figure 4). Further evidence supporting our findings showed that antioxidant supplements for 3 autistic children with oxidative stress restored low CSF 5HIAA to normal levels. In this study, two genetic defects involving serotonin reuptake proteins have only been encountered in 1 patient and identical twins with autism from consanguineous parents (first-line cousins). Our prior studies on mutations of SLC6A4, encoding SERT, and the SLC29A4 gene, encoding PMAT, in ASD suggested that normal brain serotonin homeostasis depends on adequate expression of these genes encoding the serotonin reuptake proteins SERT and PMAT, which clear serotonin from synaptic clefts and terminate serotonin signalling [22]. One child in our study carried a common SLC6A4 mutation with gain of function of the encoded SERT as previously reported [20]. In identical twins with normal CSF findings, we performed further genetic analysis because their parents were first-line cousins, and we found a SLC29A4 mutation with loss of function of the PMAT membrane transporter. However, the contribution by SLC6A4 and SLC29A4 mutations in our study remained minor with respect to the whole population of autism spectrum disorders. Another factor predisposing to low synaptic serotonin availability is the presence of low vitamin D levels, which normally interact with vitamin D responsive elements in the promotor region of the TPH2 gene and stimulate its transcription, while vitamin D simultaneously represses MAO-A and SERT gene transcription [25, 26]. Thus, low vitamin D reduces TPH2 expression and simultaneously favours higher SERT and MAO-A expression. However, with a high prevalence of vitamin D deficiency (70%) in our study, we did not confirm a correlation between plasma vitamin D concentrations and CSF 5HIAA levels (*R*^2^ = 0.0422). In only two patients with low CSF 5HIAA levels, we found a low plasma Trp level serving as substrate to the TPH2 enzyme and partly explaining lowered brain serotonin production. However, in the other patients, we did not detect low plasma Trp concentrations.

There are a few reports on low urinary Trp excretion among autistic children on a casein- and gluten-free diet as well as autistic children without a diet compared to healthy control children [52]. Other reports confirm low plasma amino acid precursors (phenylalanine, tyrosine, and tryptophan) as substrates for neurotransmitter synthesis [53]. This low Trp availability demonstrated by these reports may be explained further by the known high incidence of feeding disorders with nutritional deficiencies among children with infantile autism [20]. Therefore, care should be taken to avoid severe feeding disorders and dietary restrictions and to correct nutritional deficiencies.

Exposure to oxidative stress inhibits B_12_-dependent methionine synthase activity, which has a negative impact on the methionine cycle with reduced S-adenosylmethionine (SAM) production, resulting in failure of >100 methyl-transfer reactions. Simultaneously, oxidative stress increases the activity of cystathionine beta synthase (CBS), which shifts homocysteine away from the methionine cycle towards the transsulfuration pathway to increase cysteine and glutathione synthesis as adaptive mechanisms to protect against elevated oxidative stress. In this context, low brain MTHF availability due to FR*α* autoimmunity further inhibits methionine synthase activity, enhancing the shift from the methionine towards the transsulfuration cycle resulting in downregulation of cellular methylation capacity.

ROS also react directly with DNA purine and pyrimidine components and causes DNA strand breaks, as reflected by increased oxidized DNA damage in lymphocytes. Moreover, ROS can induce oxidative DNA damage at the level of methylated CpG islands near gene promotor sites resulting in various mutations including C ⟶ T transitions, G ⟶ T transversions, and CG ⟶ TT tandem mutations. In addition, ROS can convert guanosine to 8-oxo-guanosine and 5-methyl-cytosine towards 5-hydroxymethyl-cytosine [54–56]. These modified methyl-CpG sequences due to oxidative stress lead to functional loss of methylated CpG islands acting as recognition sites for the transcription repression machinery mediating gene silencing [56]. Thus, oxidative stress not only causes gene sequence alterations but also results in epigenetic changes with failure of methylated gene inactivation.

Because FR*α* autoantibodies block MTHF passage to the fetus and to the brain, the consequent brain MTHF depletion diminishes production of sufficient SAM as substrate of DNA methyltransferases which transfer methyl groups to CpG promotor sites that serve as recognition sites required for gene silencing. Thus, the combination of FR*α* autoimmunity and oxidative stress act independently or cooperate to derange epigenetic mechanisms controlling the orchestration of activation and inactivation of specific genes during neuronal development and differentiation [57, 58].

The consequences of folate deficiency affecting brain development may be more prominent in autistic children from mothers with folate deficiency or carrying maternal FR*α* autoantibodies during pregnancy. This study was not a treatment trial but an observational study explaining the diverse results we have obtained. Although for each parameter the results from the healthy control group did not always show a statistical difference compared to the autism group, we defined the upper and lowest boundaries for the healthy controls as the normal range. We only considered for the autism group oxidative stress biomarkers and pro- or antioxidants to be abnormal if the measured values fell outside this normal range. We found that for each individual autistic patient, pro- and/or antioxidants can have either normal values or 1, 2, or more abnormal values, where a cumulative number of these aberrant factors seems to have an impact on for example serotonin metabolism.

Among the 38 autistic patients, there was a random distribution of abnormal pro-oxidant and/or antioxidant factors without a clear association between any specific factor and the different oxidative stress biomarkers or CSF markers.

## 5. Conclusion

In the pathogenesis of low-functioning autism, oxidative stress and pre- and/or postnatal folate receptor autoantibodies appear to play an important role by affecting DNA integrity, intermediary metabolism, serotonin turnover, and one-carbon transfer mechanisms. These changes can have an impact on epigenetic programming of neurodevelopmental genes. Early detection and appropriate therapeutic intervention could reverse the core deficits and improve outcome.

## Figures and Tables

**Table 1:** Case-control study comparing oxidative stress parameters (mean ± SD) between autistic children and age-matched healthy controls.

Parameter	Autism	Controls	Reference range for controls	Statistics (*p* value)
Number	38	24		
Age (years)	7.25 ± 3.9	8.7 ± 3.2	4.4–16	*p* = 0.13
Comet assay (% oxidized DNA)	24 ± 9	14.9 ± 3.9	10.2–21.5	*p* = 0.0002
Lipid peroxides (*μ*M)	673 ± 339	609 ± 219	183–1095	*p* = 0.41
Oxidized LDL (ng/ml)	1465 ± 1377	1257 ± 1111	78–3490	*p* = 0.54
Antibodies against ox-LDL (IU/L)	747 ± 428	568 ± 419	82–1200	*p* = 0.12
Paroxonase (*μ*M/L)	147 ± 115	137 ± 75	3–282	*p* = 0.73
Copper (mg/L)	1.13 ± 0.26	0.99 ± 0.25	0.57–1.78	*p* = 0.04
Manganese (*μ*g/L)	10.1 ± 2.58	10 ± 1.86	6.98–15.24	*p* = 0.87
Selenium (*μ*g/L)	77.4 ± 21.2	77.21 ± 11.53	50.5–98.5	*p* = 0.96
Zinc (mg/L)	0.84 ± 0.16	0.84 ± 0.11	0.59–1.02	*p* = 1
Ratio copper/zinc	1.34 ± 0.35	1.17 ± 0.23	0.74–1.79	*p* = 0.04
Ceruloplasmin (g/L)	0.31 ± 0.06	0.28 ± 0.05	0.18–0.39	*p* = 0.04
Vitamine C (*μ*g/ml)	11.5 ± 4.82	14 ± 2.9	5.14–19.49	*p* = 0.027
Ubichinon Q10 (mg/L)	0.68 ± 0.25	0.64 ± 0.17	0.35–1.06	*p* = 0.50
Vitamine E (mg/L)	10.08 ± 2.17	10.7 ± 2.4	6.4–16.38	*p* = 0.30
Gamma-tocopherol (mg/L)	0.63 ± 0.41	0.70 ± 0.26	0.29–1.28	*p* = 0.46
Beta-carotene (mg/L)	0.3 ± 0.29	0.39 ± 0.25	0.05–0.99	*p* = 0.22
Thiol proteins (*μ*M/L)	402 ± 47	363 ± 67	203–436	*p* = 0.02
Total glutathione (*μ*M/L)	851 ± 204	811 ± 141	615–1162	*p* = 0.40
Oxidized glutathione (*μ*M/L)	1.24 ± 1.87	1.39 ± 2	0.53–9.37	*p* = 0.76
Glutathione peroxidase (IU/g Hb)	42.2 ± 16.5	40.25 ± 8.4	25–59	*p* = 0.60
Superoxide dismutase (IU/g Hb)	1646 ± 319	1432 ± 168	1057–1772	*p* = 0.005

The reference range for controls shows the upper and lower boundary limits for healthy controls. The determined *p* values using the unpaired student *t*-test can be found in the last column.

**Table 2:** The distribution of blocking and binding FR*α* autoantibodies in 29 children and their parents.

	P−M−	P+M−	P−M+	P+M+	Total number
Child FRab+	12	3	3	3	21*∗*(0.62 ± 0.63)
Child FRab−	5		2	1	8

Total number	17	3	5	4	29

^*∗*^Among 29 autistic children, whose parents have also been tested, 20 had blocking FR*α* antibodies associated with binding FR*α* antibodies in only 5 children. Values in parenthesis (mean ± SE: 0.62 ± 0.63) refer to blocking FR*α* antibody titers in pmoles FR*α* blocked/ml serum. In 1 child, only FR*α* antibodies of the binding type were found.

**Table 3:** CSF findings in 38 patients assessed for oxidative stress and FR*α* autoimmunity.

	Cerebrospinal fluid values					
	5HIAA↓	HVA/5HIAA↑	5HIAA + F↓	F↓	Normal	Total *N*
Ox stress+ and FRab+	8			3	7	18
Ox stress+ and FRab−	2	3	1	1		7
Ox stress− and FRab+	2			3	1	6
Ox stress− and FRab−	1	2	1	1	2	7

Number of patients	13	5	2	8	10	38

F stands for methyltetrahydrofolate (MTHF).

**Table 4:** CARS and CSF findings at baseline and following antioxidant supplementation during 8 months (patients 1 and 2) and during 2 years (patient 3) among three children with severe infantile autism.

Patient	Age (yr)	5HIAA	HVA	Ratio	MTHF	Neo	Bio	Oxidative stress	CARS
(1) Baseline	3½	104↓	566	5.4↑	117	8.6	23	Lipid peroxides↑	42
Treatment	4½	160	436	2.7	100	18	37		34
(2) Baseline	4½	81↓	338	4.2↑	95	35	19	Lipid peroxides↑	45.5
Treatment	5.3	136	495	3.6↑	113	25	61	Lipid peroxides normal	43.5
(3) Baseline	17	25.6↓	92↓	3.6↑	82.7	9.5	10.7	Ox DNA↑, Trp↓	41
Treatment	19½	80	258	3.2	49	10	7↓		
*References (median and range)*									
	2–4 yr	202 (105–299)	603 (211–871)		63–111	9–30	10–30		
	5–10 yr	133 (88–178)	523 (144–801)		41–117	9–20	10–30		
	>16 yr	103 (66–141)	261 (115–488)		41–117	9–20	10–30		

Ratio stands for the HVA/5HIAA ratio with a normal range 1.5–3.5. The sign ↑ denotes elevated value, and the sign ↓ denotes diminished value.

## Data Availability

The data used to support the findings of this study are available from the corresponding author upon request.

## Abbreviations

ADI-R: Autism Diagnostic Interview-Revised
ADOS: Autism Diagnostic Observation Schedule
B_12_-MS: B_12_-methionine synthase
BH_4_: Tetrahydrobiopterin
CARS: Childhood Autism Rating Scale
CBS: Cystathionine beta synthase
CFD: Cerebral folate deficiency
Cyst: Cysteine
FR*α*: Folate receptor alpha (*FOLR1* gene)
GTPCHI: GTPcyclohydrolase I
LDL: Low-density lipoprotein
MBP: Methyl-CpG binding protein
MTHF: 5-Methyltetrahydrofolate
MTHFR: Methylene tetrahydrofolate reductase
NO: Nitric oxide
NOS1: NO-synthase 1 (or nNOS)
NTD: Neural tube defect
PDD-NOS: Pervasive developmental disorder-not otherwise specified
PMAT: Plasma membrane monoamine transporter (*SLC29A4* gene)
RFC1: Reduced folate carrier-1
ROS: Reactive oxygen species
SAM: S-adenosylmethionine
SERT: Serotonin reuptake transporter (*SLC6A4* gene)
TH: Tyrosine hydroxylase (*TH* gene)
TPH2: Neuronal tryptophan hydroxylase 2 (*TPH2* gene)
Tyr: Tyrosine
Trp: Tryptophan.

## Supplementary Materials

Supplementary Materials Supplement 1: methods for determination of oxidative stress and pro- and antioxidants. Supplement 2: the treatment protocol based upon abnormal biochemical findings and FR autoantibodies.
