# Supplementary material for: Oxidative Stress, Folate Receptor Autoimmunity, and CSF Findings in Severe Infantile Autism
Source: Autism Res Treat. 2020 Nov 18;2020:9095284. doi: 10.1155/2020/9095284 (PMC7688371; doi:10.1155/2020/9095284)
Supplement: Supplementary Materials — Supplement 1: methods for determination of oxidative stress and pro- and antioxidants. Supplement 2: the treatment protocol based upon abnormal biochemical findings and FR autoantibodies. [file 9095284.f1.zip › 9095284.f1/Supplement I-ART.docx]

**Supplement I: Methods for determination of oxidative stress, pro- and anti-oxidants**

Blood samples were drawn in EDTA anticoagulant tubes, immediately centrifuged for 10 minutes at 3000 rpm, plasma collected and kept frozen on dry ice until parameter analysis. For vitamin C determination, 0.5 ml plasma was immediately transferred to ice-cold tubes containing 0.5 ml of 10% metaphosphoric acid. The whole mixture was frozen on dry ice. Analyses were performed by a spectrophotometric method using the reduction of 2,6-dichlorophenolindophenol (Perkin Elmer Lambda 40 Norwalk, USA) (29). Plasma vitamin E (α- and γ-tocopherols), β-carotene and ubiquinone were determined by HPLC procedure (Alliance Waters, USA) coupled with a diode array detector (PDA 2996, Waters, USA; 30) using Chromsytems kits (32000, 34000 and 68000). Thiol proteins were detected according to the Ellman’s method (31). The GSH/GSSG ratio (GSH = reduced glutathione; GSSG = oxidized glutathione) as marker of oxidative stress, was determined in whole blood by the GSH/GSSG-412 kit (Bioxytech, Oxis international Inc., Portland WA, USA). The concentration of superoxide dismutase (SOD) and glutathione peroxidase (GPx) in red blood cells was enzymatically measured by using respectively, Ransod and Ransel kits from Randox England. Activities of both enzymes were expressed as IU/g haemoglobin. The plasma levels of trace elements and metals selenium, manganese, copper and zinc were determined by inductively coupled plasma-mass spectroscopy (32). In addition we measured the levels for paroxonase, ceruloplasmin, total cholesterol and apolipoprotein B.

As a marker of oxidative damage to lipids, the analysis of lipid peroxides (ROOH) was performed with a commercial kit (Oxystat, Biomedica Gruppe, Austria). Oxidized low-density lipoprotein (LDL) in plasma samples was determined spectrophotometrically with a competitive enzyme-linked immunosorbent assay (ELISA) kit (Immunodiagnostik, Germany). The titer of free antibodies (IgG) against oxidized low-density lipoprotein (Ab-Ox-LDL) was assessed with a commercial enzyme immunoassay (Biomedica Gruppe, Austria) using Cu 2+ oxidized LDL as antigen (33). Detection of DNA damage using single-cell gel electrophoresis (Comet assay) is a versatile and sensitive method for measuring DNA damage in lymphocytes, detecting single/double-strand DNA breaks, alkali labile sites (apurinic/apyrimidinic sites), DNA cross-links, base/ base-pair damages and apoptotic nuclei in the cells. (28,34).
