# Supplementary material for: Oxidative Stress, Folate Receptor Autoimmunity, and CSF Findings in Severe Infantile Autism
Source: Autism Res Treat. 2020 Nov 18;2020:9095284. doi: 10.1155/2020/9095284 (PMC7688371; doi:10.1155/2020/9095284)
Supplement: Supplementary Materials — Supplement 1: methods for determination of oxidative stress and pro- and antioxidants. Supplement 2: the treatment protocol based upon abnormal biochemical findings and FR autoantibodies. [file 9095284.f1.zip › 9095284.f1/Supplement II_ART.docx]

**Supplement II:** The treatment protocol based upon abnormal biochemical findings and FRα autoantibodies.

**Abnormal biomarker Daily oral supplement dosage**

Zinc deficiency 0.15-0.25 mg/kg zinc-sulfate

Selenium deficiency 3-5 μg/kg sodium-selenite

Manganese deficiency 5-10 mg/kg Vitamin C, 20 IU/kg Vitamine E,

with 1 coffespoon Soya oil at night.

Manganese excess idem

Heavy metal excess (Cu, Al, Hg, Pb) idem

Raised copper/zinc ratio idem; additon of zinc supplement

Low glutathion idem

Bèta-carotene excess idem; limit foods rich in bèta-carotene

Vitamin A deficiency 600-1500 μg

Vitamin D (25-hydroxy-D) 10 μg or 400 IU

Vitamin C deficiency 5-10 mg/kg Vitamine C (maximal 500mg)

Ubiquinon-10 deficiency 2 mg/kg co-enzyme Q10

Vitamin E deficiency 20 IU/kg

Gamma-Tocopherole deficiency 1 coffeespoon soya, corn or sesame oil

Bèta-carotene deficiency Consume tomato or carot juices

Serum folate deficiency 0.5 mg/kg folinic acid

RBC folate deficiency 0.5 mg/kg folinic acid

Apolipoproteine B deficiency Supplement vitamins A D E, and vitamine K in case of

secondary coagulation disorder

FR-alpha antibodies Start with 0.5-1 mg/kg folinic acid daily;

Increase to 2 mg/kg daily without a clinical response after

six months. Maximum daily dose 50 mg.
